# A pavement distresses identification method optimized for YOLOv5s

**DOI:** 10.1038/s41598-022-07527-3

**Published:** 2022-03-03

**Authors:** Keyou Guo, Chengbo He, Min Yang, Sudong Wang

**Affiliations:** Beijing Technology and Business University, Beijing, 100037 China

**Keywords:** Computer science, Engineering

## Abstract

Automatic detection and recognition of pavement distresses is the key to timely repair of pavement. Repairing the pavement distresses in time can prevent the destruction of road structure and the occurrence of traffic accidents. However, some other factors, such as a single object category, shading and occlusion, make detection of pavement distresses very challenging. In order to solve these problems, we use the improved YOLOv5 model to detect various pavement distresses. We optimize the YOLOv5 model and introduce attention mechanism to enhance the robustness of the model. The improved model is more suitable for deployment in embedded devices. The optimized model is transplanted to the self-built intelligent mobile platform. Experimental results show that the improved network model proposed in this paper can effectively identify pavement distresses on the self-built intelligent mobile platform and datasets. The precision, recall and mAP are 95.5%, 94.3% and 95%. Compared with YOLOv5s and YOLOv4 models, the mAP of the improved YOLOv5s model is increased by 4.3% and 25.8%. This method can provide technical reference for pavement distresses detection robot.

## Introduction

Smart cities are developing with the development of artificial intelligence. Qi et al.^1^ Proposed 'privacy-aware Data Fusion and Prediction With spatial–temporal Context for Smart City Industrial Environment'. It combines smart cities with transportation and healthcare, generating data analysis and forecasting. Hu et al.^2^ proposed 'Digital Twin-assisted real-time Traffic Data Prediction Method for 5G-enabled Internet of Vehicles'. The coordinates of physical vehicles are represented on the control platform through 5G network, and reasonable transmission of their information helps optimize traffic scheduling. Xu et al.^3^ proposed “Trip Res: Traffic Flow Prediction Driven Resource Reservation for Multimedia IoV with Edge Computing”, which simplifies the complex distribution of edge servers by dividing the city map into zones and treating the edge servers within a zone as “big Edge Servers”. Wang et al.^4^ proposed “6G-enabled short-term Forecasting for large-scale Traffic Flow in Massive IoT Based on Time—Aware the Locality—Sensitive Hashing”, which was used for accurate and efficient short-term traffic prediction in large-scale IoT. Liu et al.^5^ proposed “An Attention-based category-aware GRU model for the next POI Recommendation”, which uses an attention mechanism to focus on the relevant historical check-in traces in the check-in sequence. The development of smart transportation is becoming more and more mature with the development of smart cities.

With the rapid development of road infrastructure in various countries, road inspection and daily maintenance work are increasingly heavy. The detection and repair of pavement distresses, such as cracks, cracked networks, damaged landmarks, potholes, and damaged manhole covers, become the most important part of road maintenance. Repairing pavement distresses in time can avoid road structure damage and traffic accidents. The detection of multiple types of pavement distresses has become an important research value. In the manual assessment, the technician will make the assessment according to the condition of the road, but the result of the assessment depends on the ability of different technicians. It is worth noting that this type of work requires a degree of expertise and work experience. For them, such work is boring, unsafe and expensive. In order to overcome these shortcomings, we need to adopt a lower cost and better performance of automatic pavement distresses detection technology. Automatic detection technology includes ultrasonic detection method, laser detection method, traditional computer vision detection method and deep learning detection method. In the mid-1980s, Sanasfone and Carino in the United States achieved the goal of nondestructive testing by using the mechanical wave reflection method in cement concrete and other assembled nonmetallic composite materials. Ultrasonic waves will be affected by moisture content, mix ratio, road temperature and other factors in the road surface, which lead to the fluctuation of sound velocity and thus affecting the measurement precision. Therefore, we still have a lot of research work to do. At present, a research institution in Britain is studying the automatic detection of pavement distresses by using high-speed 3D laser scanning technology. Due to the problems such as poor laser scanning speed, small data storage and weak processing ability, the technology can not realize the function of high-speed detection of fine cracks, so it has not reached the degree of engineering application. Traditional computer vision detection methods mainly include threshold-containing segmentation^6^, edge detection^7^, minimum path search^8^ and wavelet transform^9^. Machine learning methods such as handcrafted feature-based clustering^10^, random forests^11^ and support vector machines^12^ have also obtained good results in detection tasks. However, they require high-quality input images, and the detection results will be greatly affected when the external conditions change with uneven lighting and noisy interference. Therefore, in this study, we use deep learning algorithm to further explore the development of artificial intelligence to study the effect of pavement distresses detection in smart traffic.

The contributions of this study are as follows.

Real road environment is simulated, and our datasets of pavement distresses is enhanced by using our data enhancement tools.

For the specific task of pavement distresses detection, we optimize the relevant modules of YOLOv5 to make it more suitable for the detection of this task.

In the optimized YOLOv5 model, we combine some other algorithms, such as attention mechanism, CIOU algorithm and K_means algorithm, which aims to make our model more suitable for the detection of specific tasks.

Compared with the unoptimized model, this model is improved to some extent, which verifies the superiority and effectiveness of this model in the field of pavement distresses detection.

The rest of this article is organized as follows: ‘Related work’ briefly summarizes the existing work related to our task. ‘Methods’ describes in detail the work we do, including the processing of datasets, improved methods, recombination and arrangement of network models. ‘Results and discussion’ section presents and analyzes the experimental results after the deployment of our method, and discusses the advantages and disadvantages of our model. In the section of ‘Conclusions’, we summarize the whole paper and put forward the idea of improving our research in the next step.

## Related work

Artificial intelligence, as the theoretical basis of deep learning, has been updated by researchers in recent years. Liu et al.^13^ proposed A long-term memory-based model for greenhouse climate prediction. He used long short-term memory (LSTM) model to capture the dependence between historical climate data. The unreliability of label data has been studied across borders. Liu, Qi et al.^14,15^ proposed a framework for tag noise filtering and missing Tag Supplement (LNFS). They take location tags in location-based social networks (LBSN) as an example to implement our framework. In addition, They propose an attention-based bidirectional gated recurrent unit (GRU) model for point-of-interest (POI) category Prediction (ABG_poic). They combine the attention Mechanism with Bidirectional GRU to Focus on history Check-in records, which can improve the interpretability of the model.

Deep learning is gradually applied to the task of pavement distresses detection. Yusof et al.^16^ used deep convolutional neural networks for crack detection and classification of asphalt pavement images. In their study, the input to their network framework required clear and high-quality pictures with a relatively single category of predictions. This does not match the complexity of actual pavement distress. Xianglong et al.^17^ studied the recognition of road cracks based on VGG deep convolutional neural network, and the types of cracks include transverse, longitudinal and mesh. This has led to a certain increase in the variety of road diseases, but the VGG network has the disadvantage of a large number of network parameters and slow working speed, which cannot be ported to embedded devices in practical applications. In order to solve the problem of a single type of pavement disease, the number of parameters is large and the model cannot be well ported to the embedded device. V Mandal et al.^18^ proposed a deep learning framework-based pavement hazard study. He used the YOLOv5s framework for classification detection and expanded the detection sample, but the detection precision was low. Based on this, we need to further ah expand the data samples and restructure the network model to improve the detection efficiency of detecting pavement distress. Therefore, we adopt the one-stage algorithm.

The single-stage algorithm has high detection accuracy, which not only achieves success in the pursuit of high detection accuracy, but also shows excellent performance in the detection efficiency. Therefore, YOLO represents the work of the single-phase algorithm, as well as the update from YOLOv2 to YOLOv5. The YOLOv5^19^ is the latest model in the YOLO^20^ family. The network model has high detection precision and fast reasoning speed. The fastest detection speed can reach 140 frames per second. On the other hand, the weight file size of the YOLOv5s object detection network model is small, which is nearly 90% smaller than that of YOLOv4^21^, which indicates that YOLOv5 model is suitable for deploying embedded devices to realize real-time detection. Therefore, the advantages of YOLOv5s are that the network is characterized by high detection precision, light weight and fast detection speed. There are four architectures of YOLOv5, specifically named YOLOv5s, YOLOv5m, YOLOv5l and YOLOv5x. The main difference is the depth and width of the models. As there are five categories of objects to be identified in this study, the recognition model has high requirements on real-time performance and lightweight performance. Therefore, we optimize and improve the model in order to improve the accuracy and efficiency of the model.

## Methods

### Pavement distresses images acquisition

#### Materials and image data acquisition methods

**Figure 1:**
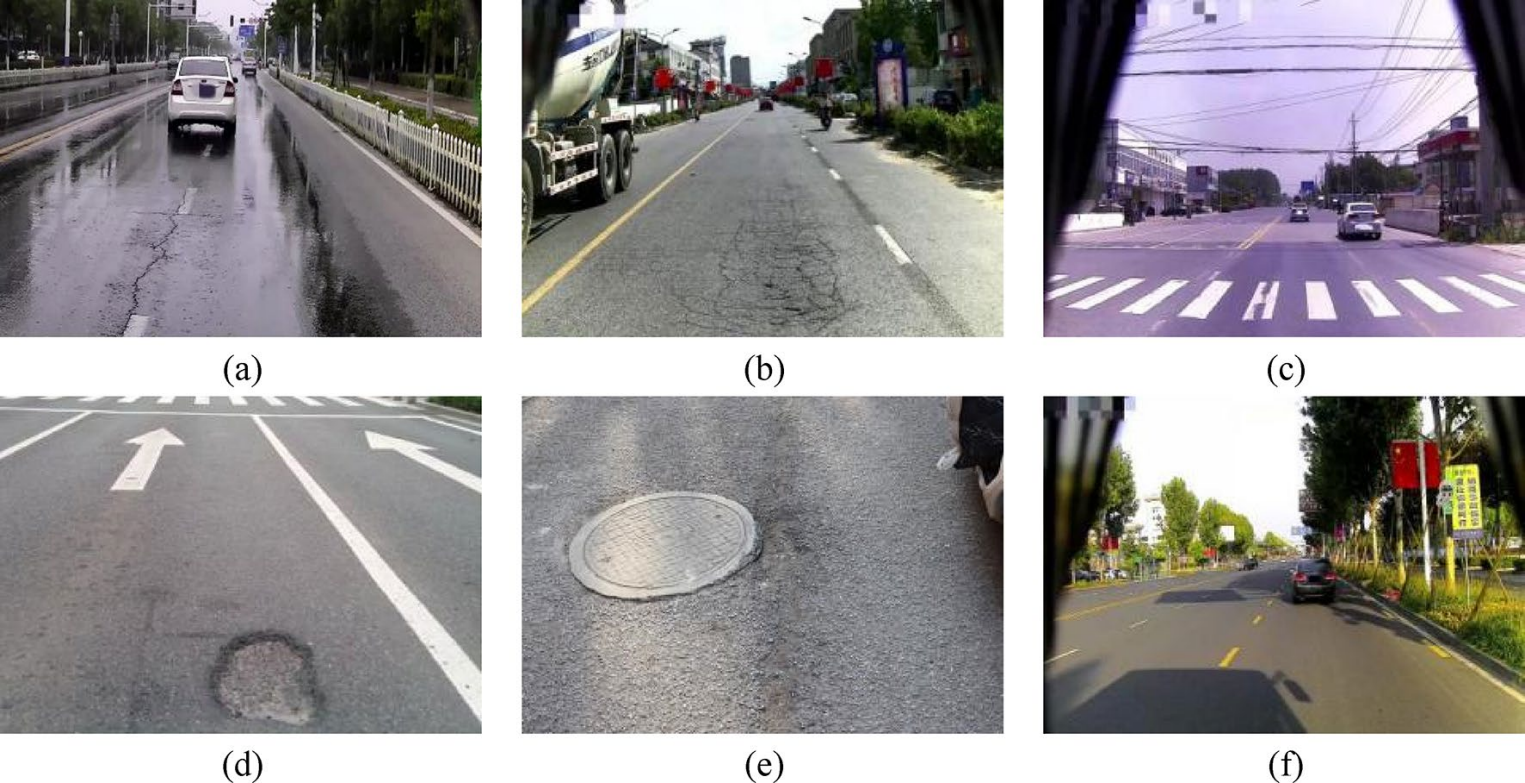
Datasets of pavement distresses under different conditions. (**a**) Crack (**b**) The cracked networks (**c**) Damaged landmarks (**d**) Pothole (**e**) Damaged manhole covers (**f**) The complete pavement under the shaded part.

The datasets we use were provided by Jiangsu Provincial Department of Industry and Information Technology. The datasets included cracks in different directions, complex cracked networks, damaged landmarks in different degrees, different potholes, damaged manhole covers, and intact roads. Figure 1 shows part of the datasets. Furthermore, the datasets were captured by an iPhone in the morning, midday, and afternoon. Shadows, rain, and other environmental conditions were considered in the shooting process. The images were captured at a resolution of 4500 pixels × 6000 pixels in jpg format.

#### Preprocessing of images

First, we randomly divided the 2338 images into two groups. One was a test datasets with 644 images, and the rest were training datasets. The detailed distribution of image samples is shown in Table 1. To improve the training efficiency of the pavement distresses recognition model, we compressed the original images of the training and val datasets so that they meet the input channel requirements of YOLOv5s. Next, Datasets labeling software "LabelImg" was used to draw rectangular boxes of multicategory pavement distresses object in the road images to achieve manual labeling of the pavement distresses. The image is labeled according to the most complete rectangle around the pavement distresses. After the annotation was completed, an XML format file was generated. Finally, to enrich the datasets, a data enhancement process was performed to better extract the features of the pavement distresses objects and to avoid overfitting the data obtained in training.

**Table 1:** Pavement distresses type comparison table.

Id	Classes	Description	Number of labels
1	Crack	Cracks	2169
2	Net	Cracked networks	370
3	Marking	Damaged landmarks,	228
4	Pothole	Potholes	471
5	Abnormal Manhole	Damaged manhole covers	1265

To improve the generalization ability of pavement distresses object detection model, a variety of image enhancement methods were applied to 1694 training datasets. The image enhancement methods include image brightness enhancement and reduction, horizontal mirroring, vertical mirroring, and multiangle rotation. In addition, we consider that the image acquisition equipment wobble will make the image blurred. In the process of datasets pretreatment, gaussian noise was added to the image and motion blur is processed. Data processing is shown in Table 2.

**Table 2:** Data enhancement algorithm.

Require: Image enhancement
1: Loading image list from file
2: Loading bounding box in all files
3: For image, bounding box in all files
4: For num in number of enhancements
5: Add noise, change lights, cut out, rotate, translation, mirror in random rates
6: Save new image and bounding box

First, Image cropping and image conversion can be implemented using functions. a new photo can be created by OpenCV ‘numpy. shape ’function. The transformed images and cropped image can improve the detection performance of the model by correctly identifying the cracks of different orientations. Second, we used OpenCV add Weighted to adjust the image brightness. Converts the original image to a blended image, which is a function that helps with alpha blending of the image. The generic syntax for this can be as follows: ‘img = cv2.addWeighted(source1, alpha, source2, beta, gamma)’. Here, we can add weights to redefine the transparency and translucency of the images. we add the image and then add the pixel values. The new image is the source where we will multiply the alpha value and the second source with the beta value. The gamma value will be added to this value and help in processing and alpha blending the image. Then, We used The flip function of OpenCV to rotate the image Angle. It has the ability to flip a two-dimensional array in different directions, whether it's vertically, horizontally, or on either axis, we can use it to do the Angle rotation of the image. And finally, the addition of Gaussian noise to the raw images was implemented using the OpenCV function ‘kimage.util. random_noise’.

**Figure 2:**
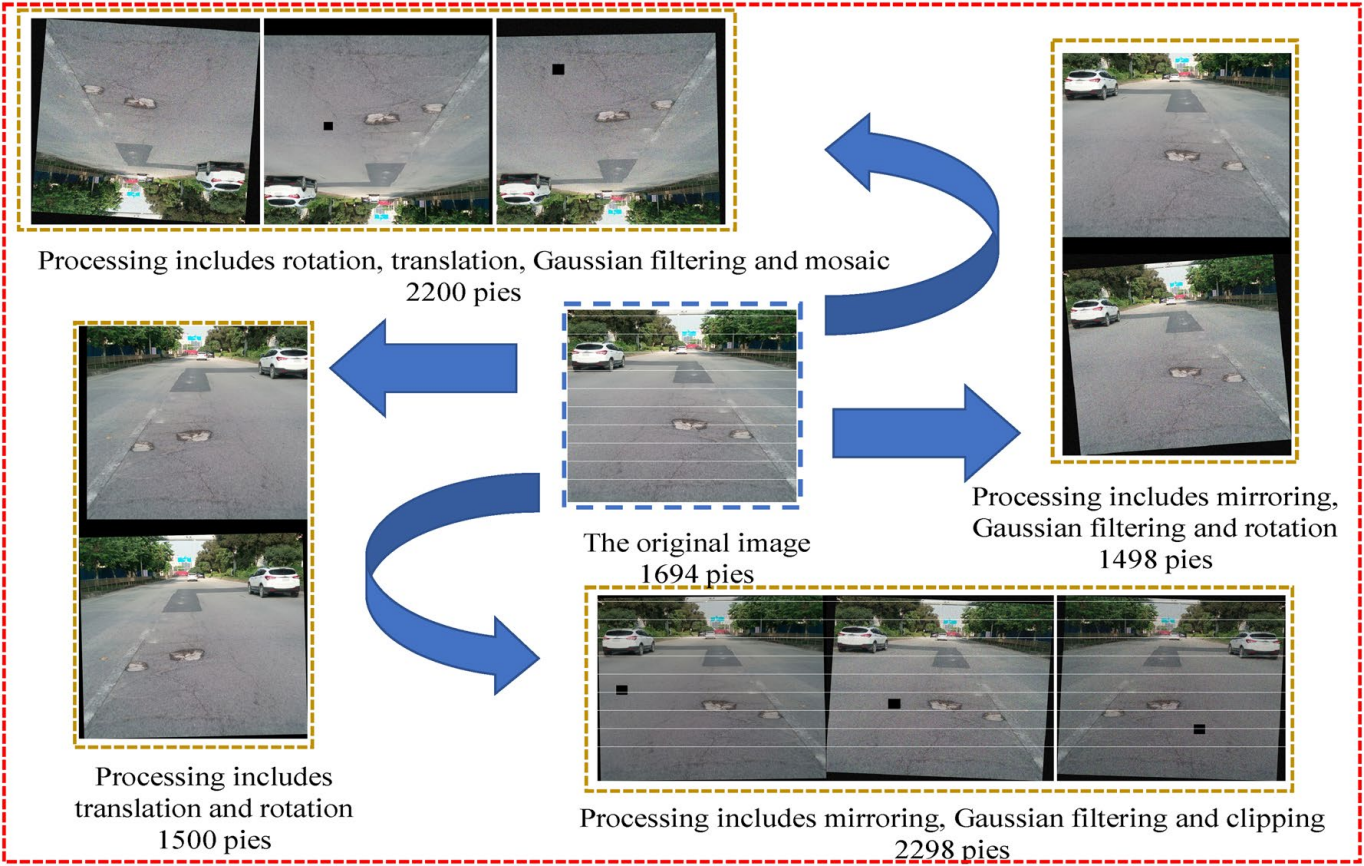
The distribution of training datasets data.

The final training datasets of the pavement distresses object recognition model consists of 9184 images, including 7490 enhanced images and 1694 images from the training datasets. It should be noted that since no pavement distresses were marked in the negative samples, they did not need to be enhanced. The detailed distribution of the training datasets is shown in Fig. 2. There was no overlap between the training and the test datasets.

### Improvement of YOLOv5s network architecture

#### YOLOv5s network architecture

**Figure 3:**
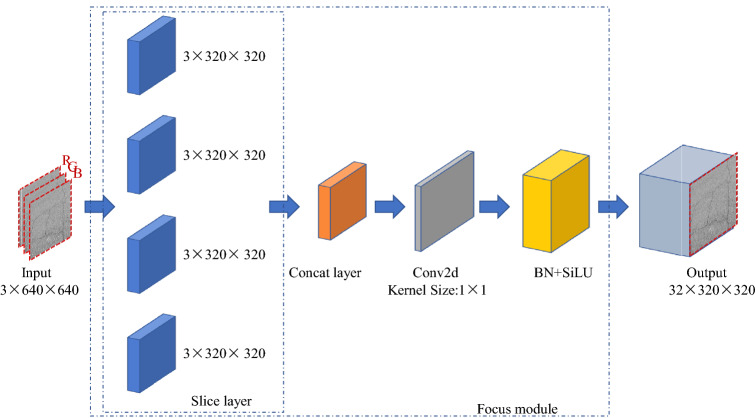
Structure of Focus module.

The YOLOv5s framework is mainly composed of three parts, namely, the backbone network, neck network, and detection network. The backbone network is a convolutional neural network that aggregates different fine-grained images and forms image features. To be more specific, the first layer of the backbone network is the focus module (Fig. 3).First, the image input with 3 channels was divided into four pieces, and its size was 3 × 320 × 320 per slice, using a slicing operation. Second, the concat operation links four slices together in depth, resulting in an output feature map of size 12 × 320 × 320. Then, through a convolutional layer composed of 32 convolution kernels, an output feature map with a size of 32 × 320 × 320 was formed. Finally, through the BN layer (Batch Normalization)^22^ and SiLU^23^ activation functions, the results were output into the next layer.

**Figure 4:**
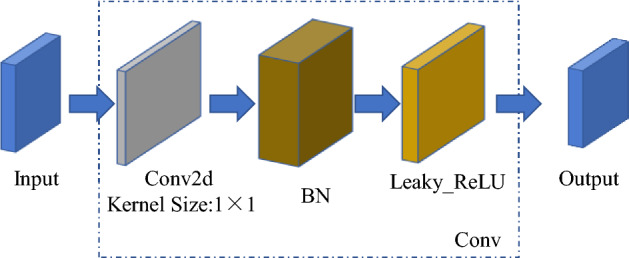
Structure of Conv module.

The second layer of the network framework is the standard convolutional layer (Fig. 4), which consists of convolutional operations, normalization processing (BN layer) and activation function Leaky_ReLU.^24^ The size of the convolution kernel is 1 × 1. The image is passed through the convolution layer to obtain the field of perception and improve the efficiency of subsequent processing.

**Figure 5:**
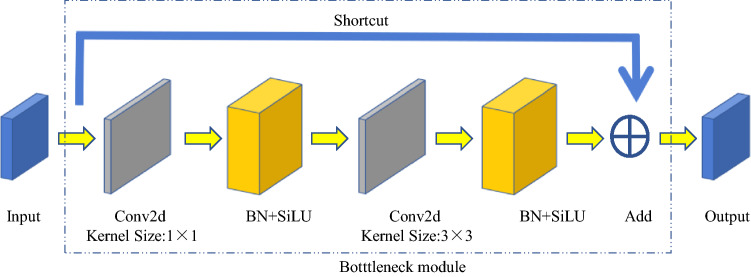
Structure of bottleneck module.

The third layer of the backbone network is the Bottleneck C3 module (Fig. 5), which aims to extract the deep features of the image better. The C3 module is mainly composed of a bottleneck module, which is a residual network architecture. C3 is composed of a convolution layer (Conv2d + BN + SiLU activation function) whose convolution kernel size is 1 × 1, a convolution layer of which the convolution kernel size is 3 × 3. The initial input and the final output of this part are added through the residual structure as the final output of the bottleneck module.

**Figure 6:**
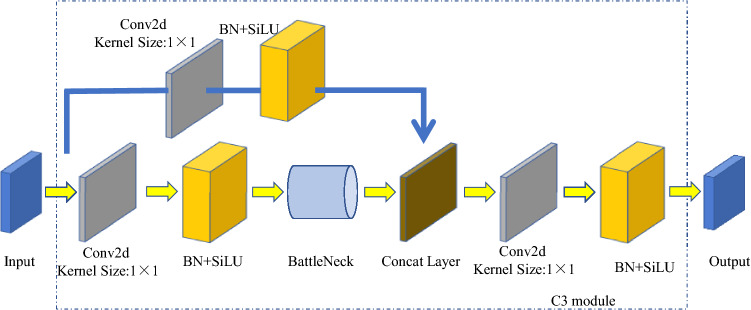
Structure of C3 module.

The C3 module (Fig. 6) input channel is divided into two branches. Through the convolution operation of the two branches, the number of channels in the feature map is halved. Then, the feature map goes through the bottleneck layer, the Con2d and BN layers in the second branch, and the concat layer is used to deeply fuse the two branches. Finally, the output feature map of the module is generated after continuous passage through the BN layer and Conv2d layer, the size of the feature map is the same as the input size of the C3 module.

**Figure 7:**
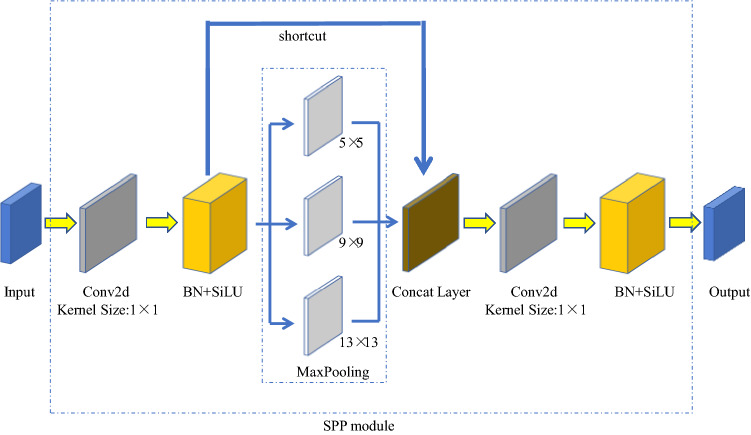
Structure of SPP module.

The SPP^25^ module (spatial pyramid pooling) (Fig. 7) is located at the ninth layer of the backbone network. It accomplishes two things: fusing multi-scale features and converting different inputs into fixed dimension vector. In the SPP module of YOLOv5s, the input feature size is 512 × 20 × 20. First, the feature map with the size of 256 × 20 × 20 is output, after a pass through the convolutional layer; the convolution kernel size is 1 × 1. Then, the feature map is subsampled under the action of three parallel max-pooling layers, and the output feature map whose size is 1024 × 20 × 20 is connected in depth. Finally, the final output feature with a size 512 × 20 × 20 is generated under the action of a convolution layer with 512 convolution kernel.

**Figure 8:**
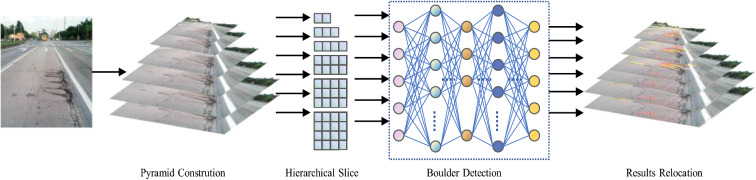
Structure of FPN module.

The neck network is composed of a certain number of mixed features and combined image features. Feature Pyramid Networks^26^ (FPN) is generated by it, then, output image feature through the detect network is generated (Fig. 8). Because the top-down FPN structure is added to the network, the feature information of the high level is transferred and fused by the subsampled method to obtain the feature map for prediction. On that basis, a bottom-up feature pyramid containing two Pixel Aggregation Network^27^ (PAN) structures has also been added behind the FPN layer. In this combination, the FPN layer transmits strong semantic features from the top to down, while the feature pyramid transmits strong positioning features from the bottom to up, the two layers work together to aggregate the parameters of different detection layers from different backbone layers.

The detection network is mainly used for the final detection part of the model. anchor boxes act on the feature map from the previous layer and output information vector, including the confidence of the objects and the maximum and minimum values of the object boundary coordinates. In the detection network of YOLOv5s, there are three detection layers, whose output feature maps are 76 × 76, 38 × 38 and 19 × 19. Each detection layer finally outputs a 30-channel vector ((5 classes + 1 class probability + 4 surrounding box position coordinates) × 3 anchor boxes). So far, the predicted anchor frames are generated, the objects in the original photos are marked, and the detection of the pavement distresses is completed.

#### Improvement of backbone network

The optimization of the network framework for the feature extraction part is based on the basic requirements of the pavement distresses recognition algorithm. On the one hand, the size of the detection model needs to be lighter, and it can be ported to hardware devices like the NVIDIA TX2. On the other hand, On the other hand, the backbone network of the model must do its best to accurately detect and identify five pavement distressess. In this study, the YOLOv5s architecture is optimized and improved. It makes it more adaptable to the task of pavement distresses detection.

**Figure 9:**
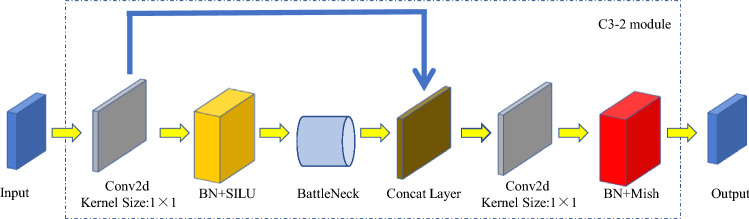
Structure of improved C3 module (C3-2 module).

The backbone network of YOLOv5s architecture consists of four C3 modules. Although the convolution operation can extract the features in the image, the convolution kernel contains a large number of parameters, which leads to the existence of a large number of parameters in the recognition model. Therefore, we optimize the C3 module in this study. The convolutional layer on the bridge branch of the original module is removed, and the input feature graph of the C3 module is directly connected with the output feature graph of another deep branch, which effectively reduces the number of parameters in the module. An activation function named Mish is added to the structure after feature fusion in order to more fully incorporate the characteristics. The architecture of the improved C3 module, named C3-2 (Fig. 9).

#### The addition of attention mechanisms

**Figure 10:**
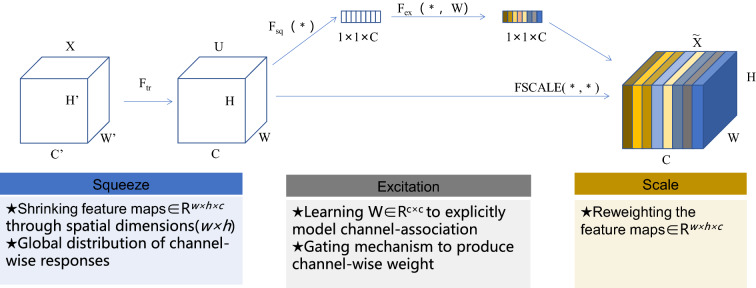
Squeeze-and-Excitation (SE) module.

We introduce the attention mechanism^28^ into the improved model. The SE^29^ module (squeeze and networks, SENET) is a network of visual attention mechanisms which is mainly used to improve the sensitivity of the model to channel features. This module is lightweight and can be applied to network architectures. It can improve the detection performance of the model with less computation. In this study, the SE module is embedded in the last layer of the backbone network to further enhance the detection effect of the improved YOLOv5s. The SE module (Fig. 10) mainly consists of two operations, squeeze and excitation, which can be applied to any mapping, and the mathematical expression of the mapping relationship is shown in Eq. (1). Taking convolution as an example, the convolution kernel is $V = [v_{1} ,v_{2} ,v_{3} ,...v_{c} ]$, $v_{c}$ denote the cth convolution kernel, output $U = [u_{1} ,u_{2} ,u_{3} ,...u_{c} ]$ from Eq. (1), as shown in Eq. (2).

1 $$Ftr:X \to U,X \in R^{H^{\prime} \times W^{\prime} \times C^{\prime}} ,U \in R^{H \times W \times C}$$

2 $$u_{c} = v_{c} *X = \sum\limits_{S = 1}^{C^{\prime}} {v^{s}_{c} *x^{s} }$$

where H and H' denote the height of the image. W and W' are the width of the image, C and C' mean the number of channels of the image, and X is the size of the input image. The ‘*’denotes the convolution operation, $v^{s}_{c}$ means the 2-D convolution kernel of a channel, and $x^{s}$ represents the picture size of the channel number. The convolution layer inputs spatial features on a channel and it learns feature spatial relations. Since the result of the convolution of each channel is ‘add’ operation, the channel feature relation is mixed with the spatial relation learned by the convolution kernel. SE module is designed to remove such confounding and make the model directly learn channel feature relations.

The SE module first performs a squeeze operation on the feature map to obtain global features at the channel level, then performs an excitation operation on the global features to learn the relationships among channels to obtain the weights of different channels, and finally multiplies the original feature map to obtain the final features. Essentially, the SE module is performing attention or gating operations on the channel dimension, and this mechanism allows the model to pay more attention to the most informative channel features and suppress those unimportant ones.

#### Improvement of initial anchor box size

The dimensions of the three feature graphs of YOLOv5s predicted network output are 76 × 76, 38 × 38 and 19 × 19. These three feature maps are multi-scale detection layers used to detect small, medium and large objects. Their initial anchor boxes sizes are ‘10,13, 16,30, 33,23’, ‘30,61, 62,45, 59,119’ and ‘116,90, 156,198, 373,326’ respectively. The original anchor boxes are only suitable for COCO datasets, but are not suitable for pavement distresses data, so samples cannot be effectively identified during testing. In order to avoid low-quality object recognition and improve the precision of object interpretation, we improve the anchor boxes in detection layer network in YOLOv5s. We use k-means algorithm to re-cluster the coordinates of anchor boxes of pavement distresses. The principle is to build K partitioned clusters according to the datasets of N given objects. Each divided part is a cluster, and the number of anchor boxes in this study is 9, so K = 9. To achieve accurate recognition of the pavement distresses object, the pavement distresses datasets is re-clustering. the anchor box is divided into ‘19,10, 51,12, 31,29’, ‘134,13, 79,30, 60,64’ and ‘291,28, 130,81, 197,140’.

#### Improvement of two loss functions

Focal loss^30^ was originally proposed by Kai ming He et al. which mainly solves the problem of model performance caused by data imbalance. The computational equations are given in Eqs. (3) and (4). Alternatively, during the training process, there are often some inaccuracies between the predicted and true values. IOU^31^ is a common metric used in object detection, whose main function is to evaluate the distance between the predicted frame and the true frame. However, there is a problem with its original definition. If two boxes do not intersect, IOU is 0. Casein this situation, the gradient is not regressed. therefore, learning and training cannot be performed. To solve these problems, Rezatofighi et al.^32^ proposed the idea of GIoU and directly set the IoU to return the value of the loss and added a measure of intersection scale using the minimum outer rectangle of the two boxes to alleviate the deficiency of IOU. However, it still has some shortcomings. Zheng et al.^33^ proposed the idea of CIoU, which takes into account the overlap area, centroid distance and aspect ratio between the prediction frame and the real frame, making the regression of the prediction frame faster and more accurate. its specific principle is shown in Eq. (5).

3 $$FL(pt) = - a_{t} \left( {1 - pt} \right)^{\gamma } \log \left( {pt} \right)$$

4 $$pt = \left\{ {\begin{array}{*{20}c} p & {if\;y = 1} \\ {1 - p} & {otherwise} \\ \end{array} } \right.$$

5 $$L_{CIOU} = 1 - IOU + \frac{{r^{2} \left( {b,b^{gt} } \right)}}{{c^{2} }} + av$$

where $a_{t}$ and $\gamma$ mean the weight coefficients. Focal loss is introduced to the improved YOLOv5s to solve the model training problem caused by sample imbalance.${\text{b}}$ and ${\text{b}}^{{{\text{gt}}}}$ respectively represent the centroids of the prediction box and the real box.$\rho$ denotes the Euclidean distance between the centroids of the two boxes, and C denotes the diagonal distance of the smallest closed rectangle that can contain both the prediction box and the real box. $\alpha ,\nu$ are used as influence factors in Formula 5, which takes into account the aspect ratio of the prediction box to fit the object box.

**Figure 11:**
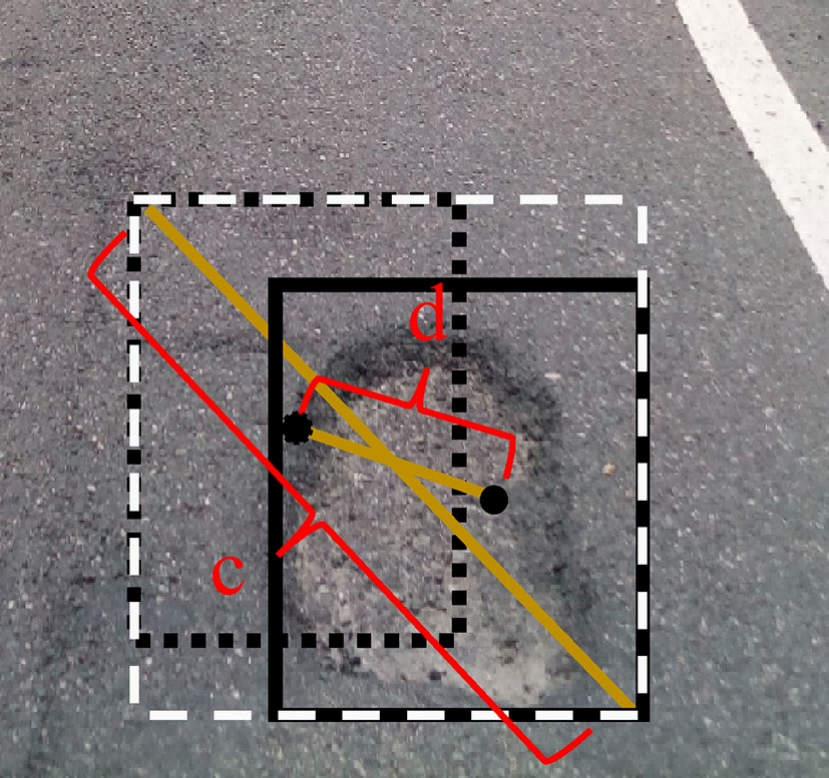
Normalized distance between the prediction frame and object frame.

As shown in Fig. 11, the box in the upper left direction represents the prediction box. The box on the lower right represents the object box. The white dashed rectangle shows the smallest outer rectangle of the prediction and object boxes. C and D represent Euclidean distances between the diagonal of the smallest outer point and the center points of the two boxes, respectively.

**Figure 12:**
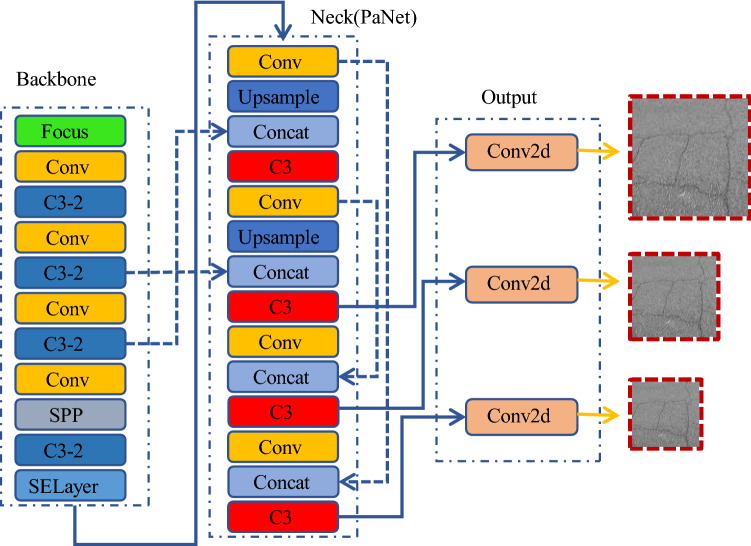
Our YOLOv5 network model structure.

We enhanced the image data and reassembled the network framework (Fig. 12). It is carried on TX2 hardware device and has good experimental results.

### Ethics approval

This study in the paper did not involve humans or animals.

### Consent to participate

All authors agree to participate.

### Consent for publication

All authors agree to participate.

## Results and discussion

### Experimental environment and settings

**Figure 13:**
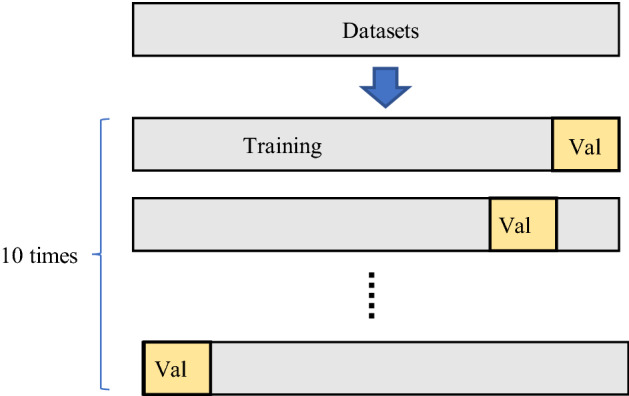
Cross-validation diagram.

Based on a personal computer (Intel(R) Xeon(R) CPU E5-2683 v3 @ 2.00 × 56,16 GB memory; NVIDIA Geforce GTX TITAN X GPU, 8 GB video memory) , the PyTorch deep learning framework was built under the Ubuntu 10 operating system in the study, and Python language was utilized to write the program code and call CUDA, Cudnn, OpenCV and other required libraries, to achieve the training and testing of the pavement distresses recognition model. In this study, the improved YOLOv5s network was trained by stochastic gradient descent (SGD) and focal loss (FL) in an end-to-end way. Considering the computing power of the graphics card we used, the batch size of the model train was set to 16. The momentum factor (momentum) was set to 0.923, and the decay rate (decay) of weight was set to 0.00951. The number of training epochs was set to 600.the anchor boxes are classified as ‘19,10, 51,12, 31,29’, ‘134,13, 79,30, 60,64’ and ‘291,28, 130,81, 197,140’. We use the same parameters to train the dataset: First, the training datasets was divided into 10 equal parts. In the second step, every nine equal parts were randomly trained as training datasets, and the training was repeated 10 times. The schematic diagram of the training method is shown in Fig. 13. The average parameters of 10 experiments were used as the final model performance index. After the training is complete, save the weight file for identification. The val datasets that did not participate in the training for the 10 times were used for testing. The final results were obtained, including precision, recall and mAP.

### Results and analysis of road damage object detection

**Figure 14:**
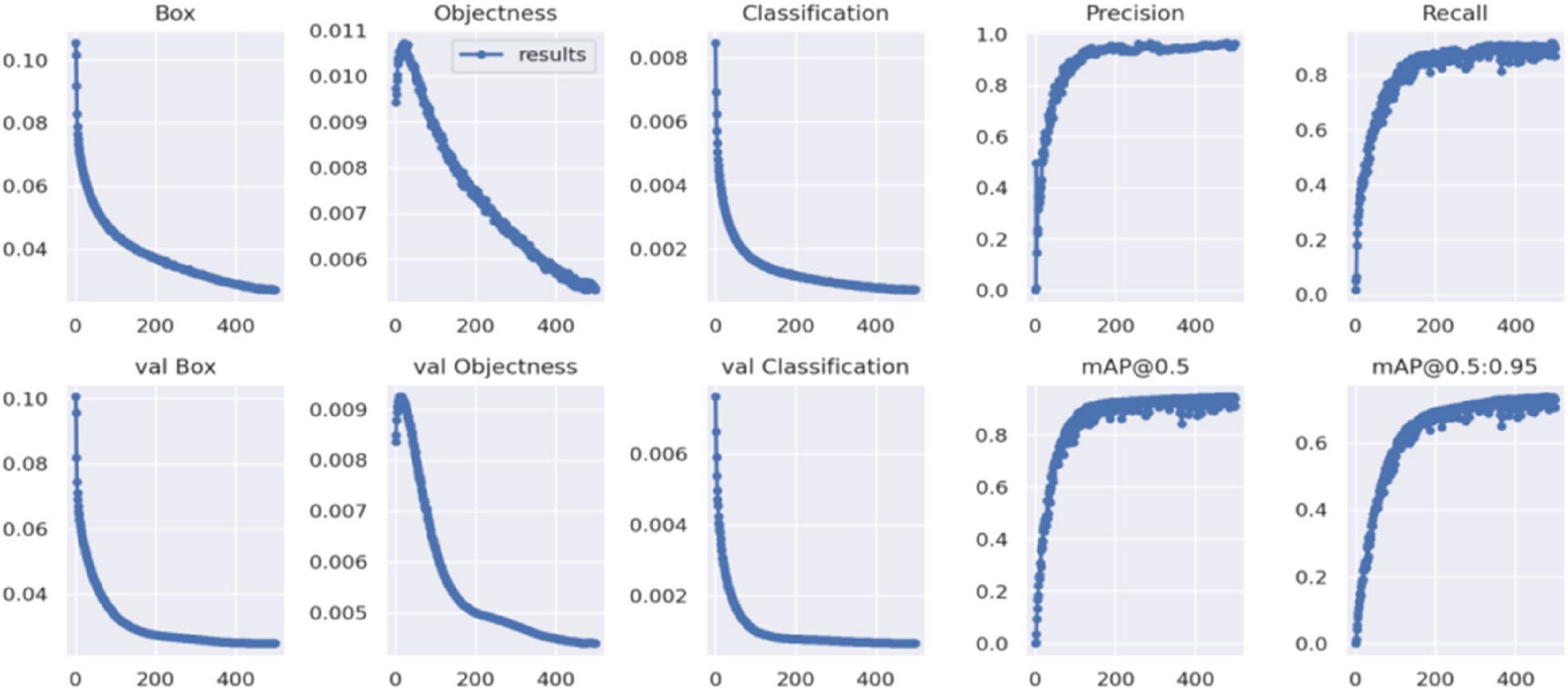
Result graph.

To judge the model training process and the effectiveness of object detection, In this experiment, box loss, objectness loss, classification loss, precision, recall, mAP@0.5, mAP@0.5:0.95 of the training and test datasets are used as the main parameters to judge the degree of convergence. For the six loss functions (val) box loss, (val) objectness loss, and (val) classification loss, the smaller the value of their parameters, the better the training effect. The training result (Fig. 14) show the relationship between the six types of loss functions and epoch, correctness and epoch, recall and epoch, and mAP value and epoch. The loss values drop rapidly in the first 300 epochs of network training and stabilize after 450 periods.

The results in Fig. 14 show that the model is not overfitted. Among the six types of loss functions of ‘box’, ‘objectness’, ‘classification’, ‘val box’, ‘val objectness’ and ‘val classification’, Each type of loss function decreases as the "epoch" of the training and test datasets steadily decreases. In addition, the test datasets does not show a decrease followed by an increase, which indicates that we have selected the "Epoch" parameter within a reasonable range. In the field of object detection, the objective evaluation of the model is precision, recall and mAP, and the formulas are calculated in Eqs. (6) to (8).

6 $$Precision = \frac{TP}{{TP + FP}}$$

7 $$Recall = \frac{TP}{{TP + FN}}$$

8 $$mAP = \frac{1}{C}\sum\limits_{K = i}^{N} {P({\text{k}})\Delta R({\text{k}})}$$

where TP (true positive) denotes the correct identification of pavement distresses such as cracks, cracked networks, damaged landmarks, potholes, and damaged manhole covers. FP (false negative) means the misclassification of actual pavement distresses. FN (false negative) denotes unidentified pavement distresses. C expresses the number of Pavement Distresses object categories. N denotes the IOU number of thresholds. K denotes the IOU threshold. P(K) denotes precision, and R(K) denotes the recall rate.

**Figure 15:**
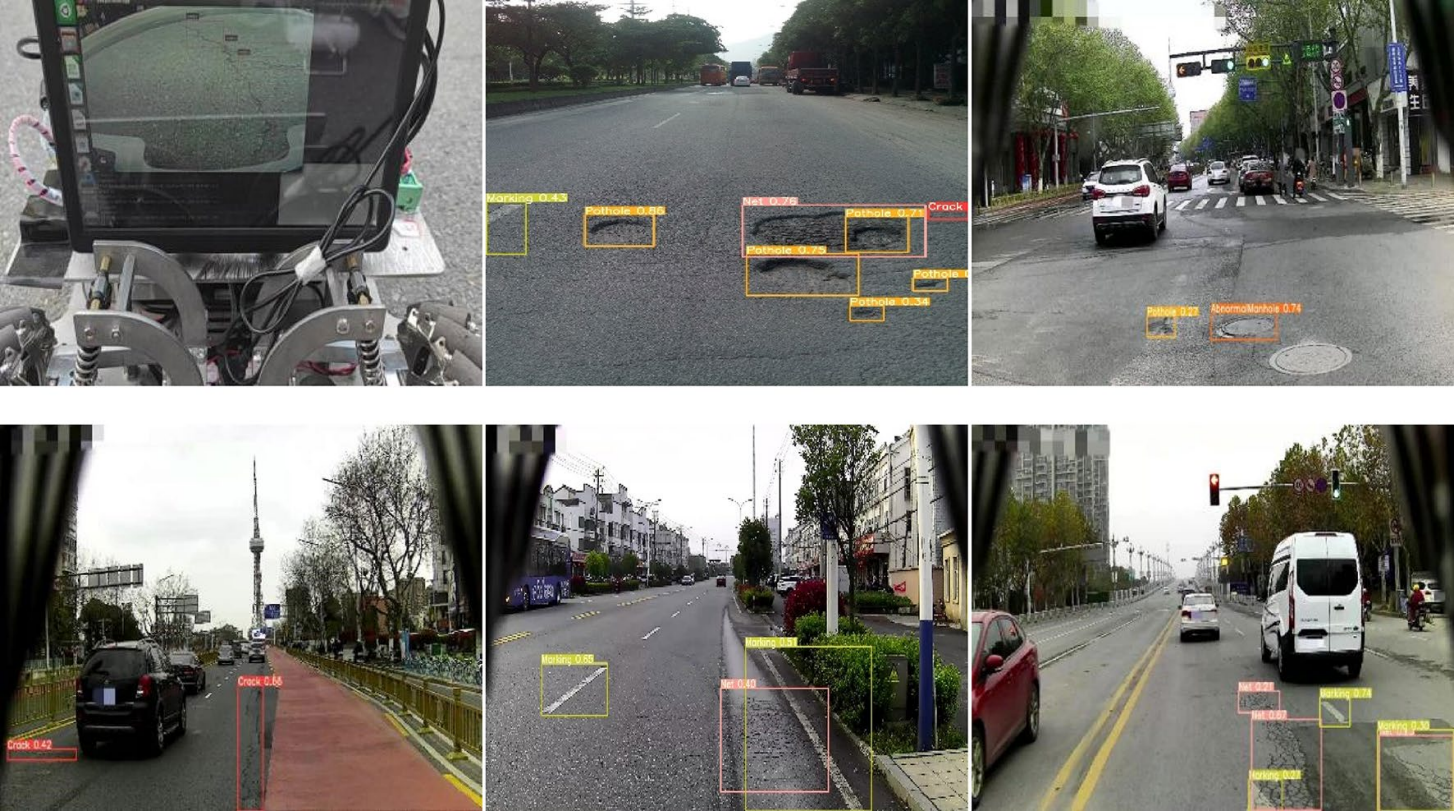
The deployment of the improved YOLOv5s in a self-built mobile platform and its object recognition results.

Through comparison and discussion of 5 improved methods, we verified that the improved methods had a positive impact on the performance of YOLOv5s model. The impact of different methods on model performance is shown in Table 3. "√"indicates that the improved policy is used in the network experiment, and “—” indicates that the improved policy is not used. The improved YOLOv5s has a good performance in road surface distresses detection on the self-built mobile platform (Fig. 15).

**Table 3:** Results of object recognition using the improved YOLOv5s network.

Method	C3_2	SE	Data Enhancement	K_means algorithm	Loss function	mAP (%)
YOLOv5s	—	—	—	—	—	90.7
Improvement I	√	—	—	—	—	89
Improvement II	√	√	—	—	—	93
Improvement III	√	√	√	—	—	94
Improvement IV	√	√	√	√	—	94.3
Improvement V	√	√	√	√	√	95

**Figure 16:**
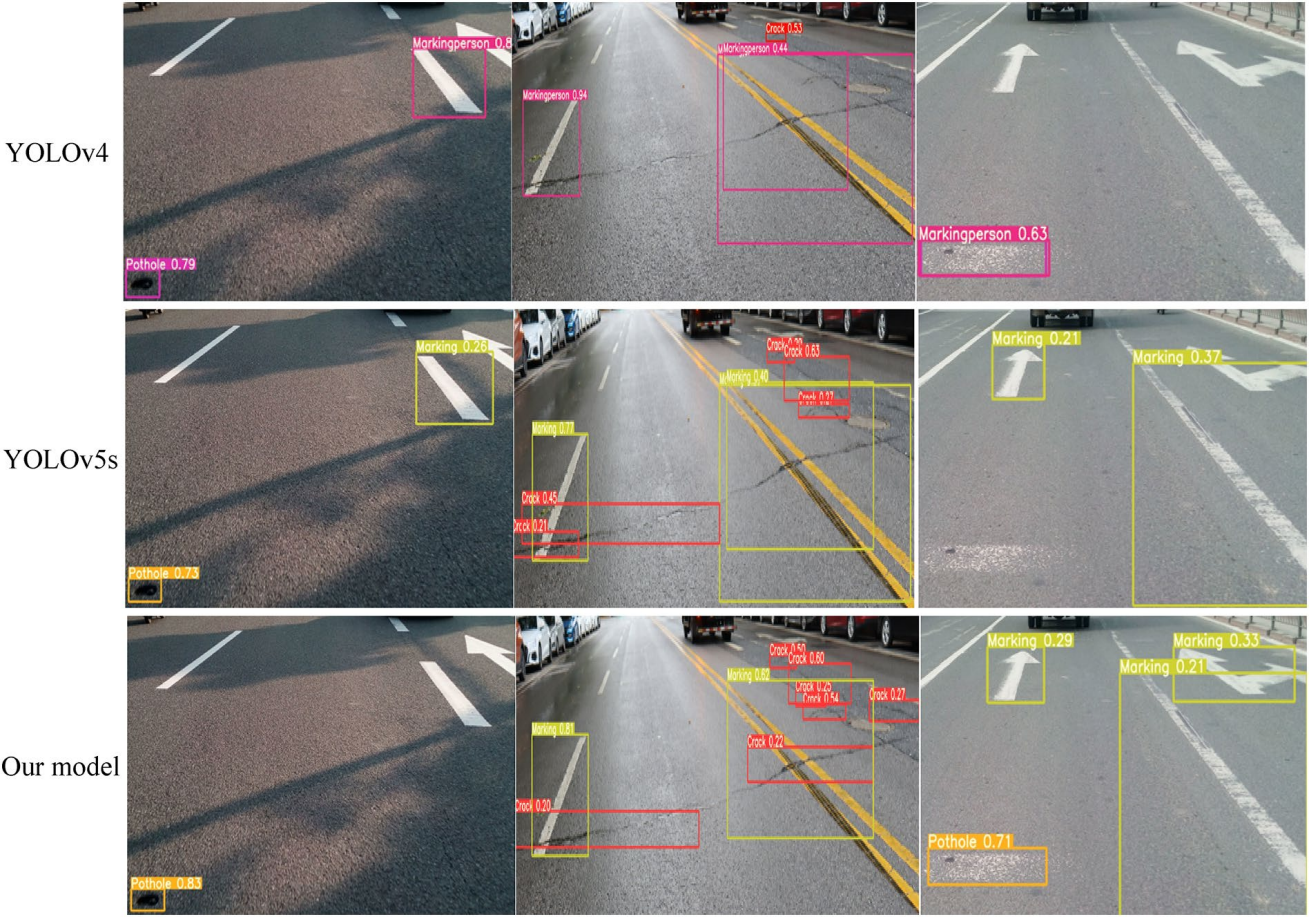
Object recognition results of the improved YOLOv5s network.

To verify the feasibility of this research method in practical applications, the optimized YOLOv5s are compared with YOLOv5s and YOLOv4 (Fig. 16). The improved detection precision has some improvement compared with YOLOv5s and YOLOv4. YOLOv5s and YOLOv4 have some errors in classification, such as identifying the repaired road surface as cracks and misidentifying the intact pavement markings ground road signs without damage defacemen, etc. In addition, the precision and recall rate of YOLOv5s and YOLOv4 are lower than the improved model.

**Figure 17:**
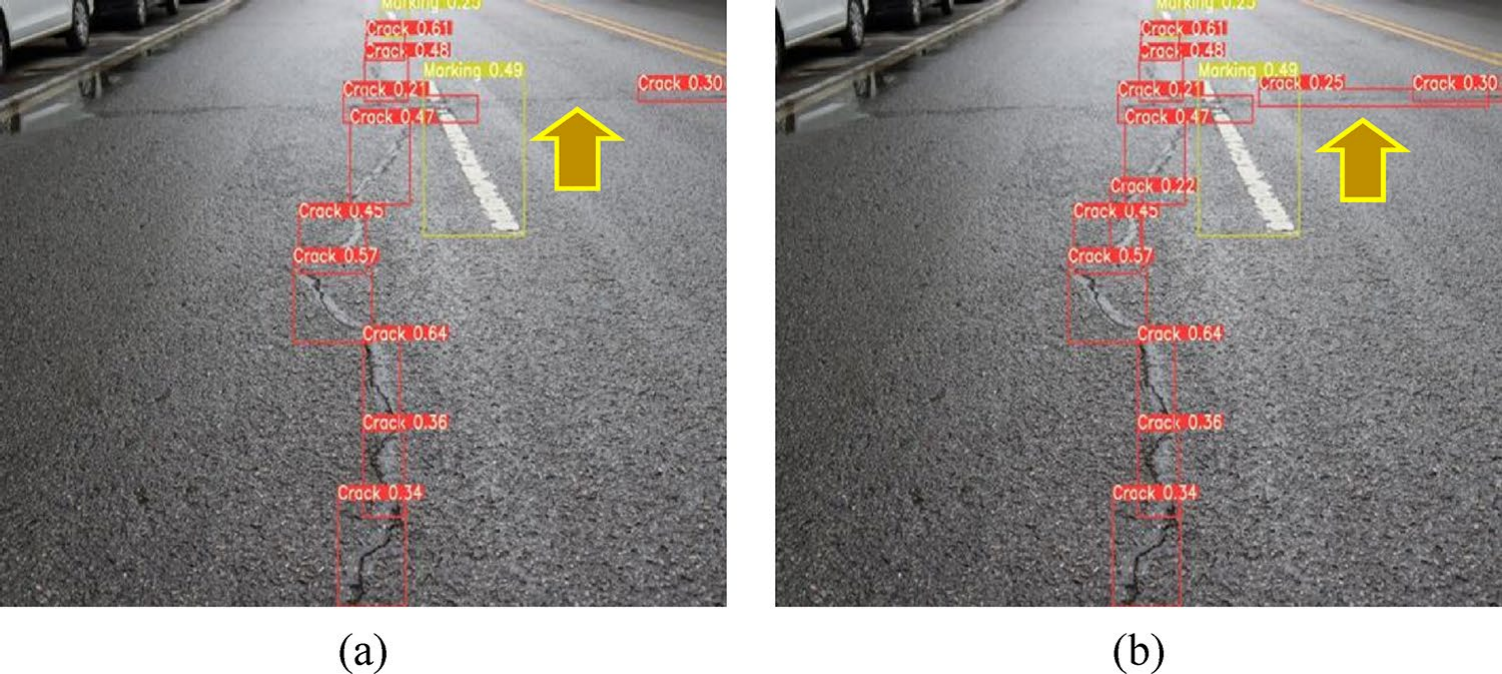
Impact of confidence threshold on detection result. (**a**) Threshold is not appropriate (**b**) Threshold is appropriate.

The identified network model needs to be predicted after training and testing. In the prediction process, the confidence value is first carried out from high to bottom, and then the prediction object is screened through CIOU. It is worth noting that based on the same network framework, different prediction confidence thresholds, detection accuracies, and recall rates is different. If the set threshold was inappropriate, the predicted result is as shown in Fig. 17. The elongated cracks is not correctly identified.

**Figure 18:**
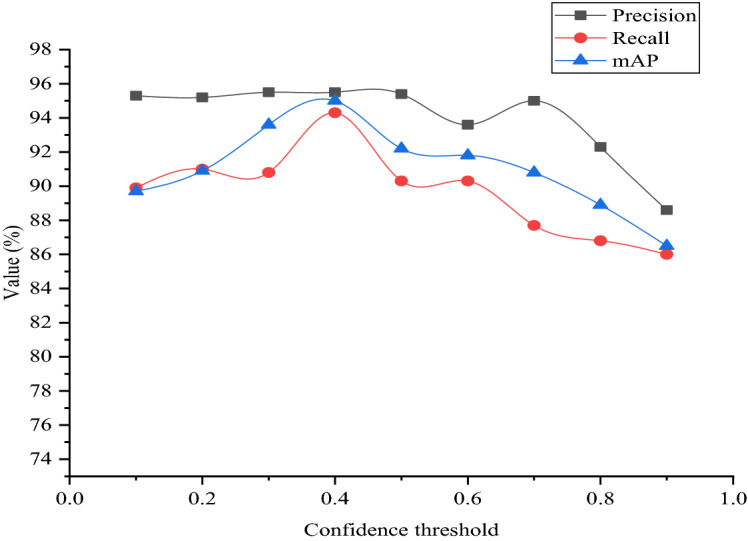
Numerical changes in various parameters under the model.

In the task of pavement damage detection and recognition, we need to select an appropriate confidence threshold, which will make the detection effect more consistent with the actual situation. By testing the test datasets, we set different thresholds to compare precision, recall, and mAP. The results of the test are shown in Fig. 18.

As can be seen from Fig. 18, when the confidence is above 0.6, the precision, recall and mAP all show a downward trend. When the confidence is between 0.3 and 0.5, all the three values have good effect. Therefore, considering the model's recognition precision, recall, and mAP, the model performs best when the confidence is set to 0.4. Precision, recall and mAP are 95.5%, 94.3% and 95%.

This study compares the optimized YOLOv5s model with the Paper 18 method and other mainstream object detection model frameworks in terms of precision, recall and mAP values. The results are shown in Table 4. Based on paper 18, we extend the data sample and optimize the YOLOv5s network model to adapt to the embedded intelligent mobile platform. In paper 14, Yuwen Liu et al. improved the robustness of the model by using the mechanism of increasing attention. In this study, we also introduce the attention mechanism in the CV field into the optimized model. These optimization methods improved the detection efficiency of our model by 4.3% overall. The proposed algorithm is a lightweight pavement distresses detection algorithm that can be installed on an intelligent mobile platform. It can divide pavement distresses into various types to meet the actual needs. In addition, its weight file size is 16 MB, and the recognition speed of each image is 0.003 s (32 FPS), which meets the requirements of real-time object recognition.

**Table 4:** Comparison of current mainstream object detection algorithms.

Approachs	Precision (%)	Recall (%)	mAP/AP (%)
Paper^18^	59.021	57.296	58.14
Faster R-CNN	38.3	41.2	39.5
YOLOv3	53.6	58	55
YOLOv4	65.3	72	70.8
YOLOv5s	94.8	90.2	90.7
Our model	95.5	94.3	95

Our model has a lot of flexibility. Users can choose the right size of the network system for research and development according to the actual situation. In this study, considering the actual application environment, we chose the smallest YOLOv5s network as the basic model and installed it on the NVIDIA®Jetson™TX2 device to enable real-time detection of multiple types of pavement distresses objects. But the algorithm still has some limitations. First, we only discuss five types of pavement distresses types. In fact, this does not fully meet the actual demand. we need to further expand the sample size in the next step. Second, when we use this algorithm to detect the category of rutting, the rutting is often incorrectly identified as crack or pothole. This is because the characteristics of ruts are more similar to large pits. To a certain extent, human observation can also lead to false identification. Third, although the algorithm has taken into account environmental factors such as rain, sunshine, and cloudy days, the detection of the object is not ideal in the nighttime state, especially when the smart car is in motion. These problems need to be solved further. Overall, the improved network model has the following advantages: first, it can detect more pavement distresses; second, it is suitable for the loading of intelligent mobile robots; finally, the proposed model is relatively light, indicating that more equipped equipment can be selected to reduce the hardware cost of computer vision.

## Conclusions

In this paper, we propose a pavement distresses identification method optimized for YOLOv5s to detect multiple types of Pavement distresses under different conditions. In the proposed method, first, we expand the data sample of the study. Second, the original C3 module is improved in Backbone network and YOLOv5s is improved by using K_means algorithm and loss function. Finally, we combined the attention mechanism with the improved YOLOv5s algorithm to obtain our own model. Compared with other algorithms, our own model can improve the precision of object detection. At the same time, it is feasible to deploy the algorithm in self-built intelligent mobile robot. In the future, we will further increase the location information of the detection object, so that the information can be transmitted to the computer in real time.

## Data Availability

Data or code presented in this study are available on request from the corresponding author.
